# Antipsychotic prescribing practices in real-life (APPREAL study): Findings from the French National Healthcare System Database (2007–2017)

**DOI:** 10.3389/fpsyt.2022.1021780

**Published:** 2022-10-31

**Authors:** Benjamin Rolland, Faustine Dalon, Noémie Gauthier, Mikaïl Nourredine, Marjorie Bérard, Louise Carton, Georges Brousse, Pierre-Michel Llorca, Flore Jacoud, Eric Van Ganse, Manon Belhassen

**Affiliations:** ^1^Centre Hospitalier Le Vinatier, Hospices Civils de Lyon, Academic Department of Addiction Medicine (SUAL), Bron, France; ^2^Université Claude Bernard Lyon 1, Lyon, France; ^3^PELyon, Lyon, France; ^4^Saint-Cyr au Mont d’Or Hospital, Hospital Pharmacy, Saint-Cyr-au Mont-d’Or Psychiatric Hospital, Saint-Cyr-au Mont-d’Or, France; ^5^Hospices Civils de Lyon, Pharmacotoxicology Laboratory, Department of Clinical Research and Epidemiology, Lyon, France; ^6^Faculté de Médecine Lyon Sud, Lyon 1 University, Lyon, France; ^7^CHU Lille, Department of Pharmacology, Inserm, Lille Neuroscience and Cognition, UMR-S1172, Université de Lille, Lille, France; ^8^CMP-B CHU, CNRS, Clermont Auvergne INP, Institut Pascal, University Clermont Auvergne, Clermont-Ferrand, France; ^9^Respiratory Medicine, Croix Rousse University Hospital, Lyon, France; ^10^Claude Bernard Lyon 1 University, Research on Healthcare Performance (RESHAPE), INSERM U1290, Lyon, France

**Keywords:** antipsychotics, France, prescribing, adherence–compliance–persistance, pharmacoepidemiogy, polymedication

## Abstract

**Background:**

Antipsychotics are used in a large variety of psychiatric and neurological disorders; investigating their use in real life is important to understand national prescribing practices, as well as to determine the levels of patient adherence.

**Methods:**

Using a 1/97e random sample (General Sample of Beneficiaries, EGB) of the French health insurance reimbursement database, we conducted a historical cohort study on the 2007–2017 period. The aim was to describe the sociodemographic characteristics of patients, the types of antipsychotics dispensed, the types of prescribers, the mean doses and average durations of treatment, the co-dispensed medications, and the levels of adherence to treatment. To exclude punctual uses of antipsychotics, we selected only patients with a continuous dispensing of the same antipsychotic over at least 3 months.

**Results:**

In total, 13,799 subjects (1.66% of the EGB sample) were included (56.0% females; mean age 55.8 ± 19.4 years). Risperidone (19.3%), cyamemazine (18.7%), olanzapine (11.9%), tiapride (8.8%), and haloperidol (7.5%) were the five most prescribed antipsychotics. 44.9% of prescriptions were written by general practitioners, 34.1% by hospital practitioners, and 18.4% by private-practice psychiatrists. On average, the mean dispensed doses were relatively low, but the variation range was large. Long-acting forms were used in 5.4% of the sample, and clozapine in 1.3%. 34.2% of patients received more than one antipsychotic, and almost 15% were prescribed at least three concomitant antipsychotics. Paliperidone and clozapine were associated with the highest levels of adherence, and risperidone and haloperidol with the lowest ones.

**Conclusion:**

An important heterogeneity of antipsychotic prescribing practices was observed in France. The rate of use of long-acting antipsychotics was low, whereas multiple antipsychotic prescriptions were frequent.

## Introduction

Antipsychotics constitute a heterogeneous set of therapeutic drugs, whose main indications consist in treating psychotic symptoms or stabilizing mood in chronic and severe mental disorders, such as schizophrenia, schizoaffective disorder, bipolar disorder, or severe depression (1). Antipsychotics are also approved in autism and Tourette’s syndrome; they are commonly used without approved indication in some other neurological or psychiatric disorders, in particular to diminish behavioral complications of dementia (1). Some antipsychotics have specific national indications: in France, cyamemazine is approved for acute or long-term treatment of anxiety or aggressiveness (2), while tiapride is indicated as a short-term treatment of agitation or aggressiveness (3). Besides official indications, other antipsychotics, such as quetiapine, may be used off-label with lower scientific evidence to treat unspecific symptoms such as insomnia or anxiety (2). Moreover, in France, while levomepromazine is not officially approved for the long-term treatment of agitation or aggressiveness, it is frequently used in the same way as cyamemazine. Last, outside the scope of neurological or psychiatric disorders, antipsychotics may be prescribed to treat headache (3) or cancer-related nausea (4), although rarely in the long term.

In addition to this great variability in the indications for antipsychotic use, an extreme heterogeneity has also been observed between countries in the overall frequency of antipsychotic use, as well as in the types of molecules prescribed. For example, a large international study comparing the prescribing practices in 16 countries found that the global rate of persons treated with antipsychotics varied from 2.8 to 78.9/1,000 in the adult general population (5). Moreover, while quetiapine, risperidone, and olanzapine were the most frequently used drugs, the types of antipsychotics prescribed were actually very heterogeneous, which can be observed through important features, such as the ratio of first-generation (FGAP) vs. second-generation antipsychotics (SGAP), or the frequency of use of long-acting forms. Such between-country disparities can result from national differences in the approval, marketing, and availability of some medicines, but they can also reflect more or less durable national habits among prescribers. These national idiosyncrasies can apply to approved indications or be observed in off-label uses of antipsychotics, which are common and may apply to indications, doses, or drug combinations (3, 6, 7).

In France, some previous studies have explored the prescribing patterns of antipsychotics in the general population. The first of these studies (8) was conducted in the French General Sample of Beneficiaries (EGB), which is a 1/97th random sample of the French health insurance reimbursement database. In this 8-years-long (2006–2013) historical cohort study, the authors explored the rate of antipsychotic prescription in the general population, including in children and adolescents, as well as the evolution in the SGAP/FGAP ratio, and the co-prescription of psychotropic drugs. They found that the prescription rate was relatively stable over time, that is, approximately 20/1,000 in the general population; the prescription rate substantially increased in children and the SGAP/FGAP ratio also increased constantly throughout the study period. Moreover, this study found that the co-prescribing of psychotropic drugs was very frequent, what raised possible safety issues. A more recent study was conducted using the EGB, but only for the year 2015; the authors found that the rate of persons receiving at least one antipsychotic prescription was 21.9/1,000, and that the SGAP/FGAP ratio was 1.02 (9). The study also provided a succinct distribution of the individual molecules in the prescription rate, but did not explore the specialty of prescribers, the average dosage and lengths of treatment, or the level of patient adherence. Other studies that explored the prescribing patterns of antipsychotics in France were focused on specific disorders, such as schizophrenia or dementia, and/or did not encompass office-based practice (10–12).

In the “*Antipsychotic Prescribing Practices in Real-life”* (APPREAL) study, we aimed to describe, using the EGB, the sociodemographic characteristics of patients, the types of antipsychotics dispensed, the types of prescribers, the mean doses and average lengths of treatment, co-dispensed treatments, and adherence to treatment among subjects treated for at least three consecutive months with antipsychotics between 2007 and 2017.

## Materials and methods

### Study design and data sources

This was a historical cohort study conducted using the EGB database. The EGB records anonymous individual information from primary and secondary care (data from PMSI, the French diagnosis-related group-based medical information system); it currently covers more than 98% of the French population. It contains: (a) characteristics (gender, month and year of birth, month and year of death if applicable), free-access-to-care status (100% of healthcare expenses are covered for individuals whose financial resources are below a set threshold), residence, chronic disease status (ALD) (patients with a registered ALD benefit from full coverage for all medical expenses related to the chronic disease); (b) all non-hospital reimbursed healthcare expenditures with date and code (medical visits and procedures, laboratory tests, drugs, and medical devices, but not the corresponding medical indication or results); (c) hospital discharge summaries (ICD-10 diagnosis codes for all medical, obstetric, and surgical hospitalizations with the date and duration of hospitalization, medical procedures, hospital department, and cost codes); d) information on prescribers (hospital or private practice and specialty for those in private practice (13, 14).

### Study population

The study population consisted of all patients aged ≥ 18 years who were reimbursed for the same antipsychotic over at least 3 consecutive months (1 per calendar month) between January 1, 2007 and December 31, 2017. To ensure analytical data exhaustivity, patients not continuously covered by the French national Health Insurance provider over the 12 months preceding inclusion and over the follow-up were excluded, as were patients with less than 12 months of follow-up.

The inclusion date was that of the first of the three consecutive dispensings of at least one antipsychotic drug. If more than one antipsychotic (excluding cyamemazine and levomepromazine) were dispensed over at least three consecutive months, the “primary antipsychotic” (PAP) was defined as the one dispensed for the longest period of time. Cyamemazine was only considered a primary antipsychotic when it was dispensed, because its official indication and practical use in France pertain to reducing anxiety and aggressiveness, but not to diminishing psychotic symptoms. If more than one antipsychotic (excluding cyamemazine) were dispensed over the same time period, the primary antipsychotic was the one dispensed first. When these criteria did not allow the primary antipsychotic to be defined, an expert group (BR, NG, MN, GB, and LC) was consulted to make a decision.

Selected patients were followed from the inclusion date until the end of follow-up, which was defined as: last health record (i.e., last care recorded in the database prior to a 6-month period without any reimbursed career related to antipsychotics), date of death, or end of the study period (i.e., December 31, 2018), whichever occurred first.

### Study outcomes and variables

Comorbidities were identified within the 12 months before inclusion, based on chronic disease status or hospital diagnoses. The following variables were described for each PAP: (1) prescriber’s specialty, (2) number of units dispensed during the first 12 months of treatment, (3) total time on treatment (defined as the time between the first and the last dispensings of the PAP), and (3) number of co-dispensed antipsychotics.

The level of non-adherence was estimated by the non-exposure to the PAP, using the percentage of Days without Treatment (%DwT)” (15), which was calculated over the 12 months following inclusion. It was assumed that each dispensing covered 30 days, regardless of the quantity or dosage dispensed. Thus, theoretical dates for the antipsychotic dispensings were calculated as the date of the first dispensing plus 30 days. The number of days between the theoretical and the actual dispensing dates was calculated to estimate the patient’s antipsychotic-free period. If the PAP was dispensed after the theoretical dispensing date, the % DwT was incremented by the number of days between the theoretical and the actual dispensing dates. If the PAP was dispensed before or on the same day as the theoretical dispensing date, the % DwT did not change. The % DwT could not be of 100% because patients were included only if they received at least three dispensings. As a result, its theoretical maximum value is 75%.

The average dispensed daily dose was calculated as follows: for each PAP, the number of units dispensed between the inclusion date + 1 month and the date of the last dispensing (within a 12-month window) was multiplied by the number of mg per unit, and this result was divided by the number of days between the inclusion date + 1 month and the last dispensing. We chose not to take into account the first month after inclusion because the dosage in the first weeks of treatment is often adjusted according to the patient’s response.

### Statistical analyses

Socio-demographic and clinical characteristics were described with descriptive statistics as follows: for quantitative variables, the sample size (N), mean, standard deviation, median, and interquartile range (IQR) were reported, and for qualitative and ordinal variables, the sample size (N) and the frequency were reported. The presence of the PAP dispensed in pharmacy was assessed during the 12 months preceding inclusion. The number and percentage of patients who received each PAP is reported. The average time on the PAP, average duration between two dispensings, and the number of units dispensed were described using mean, standard deviation, median, quartiles, minimum and maximum.

The distribution of the % DwT was described using mean, standard deviation, median, quartiles, minimum and maximum. As the % DwT was calculated over 12 months, patients changing treatment during this period would have an overestimated % DwT. As such, the % DwT was described for patients treated with the PAP for at least 12 months. The dispensed daily dose was expressed in mg per day and described using mean, standard deviation, median, minimum and maximum. All statistical analyses were performed using SAS (SAS Institute, North Carolina, US), version 9.4.

## Results

### Study population

**FIGURE 1:**
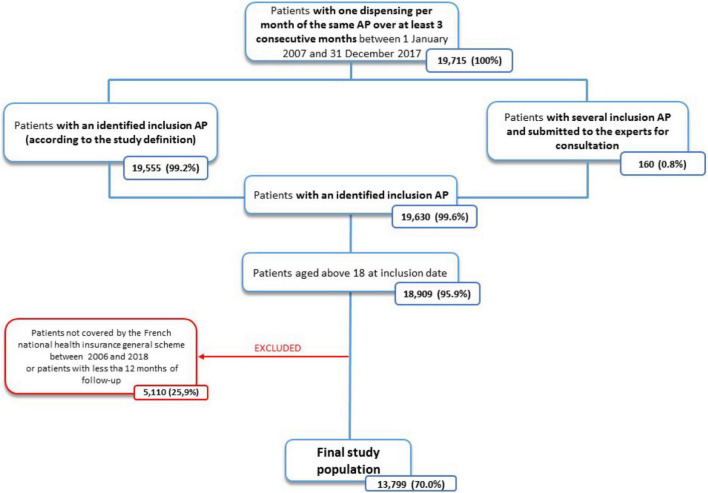
Flowchart of the study population.

This study included 13,799 individuals that met the inclusion criteria (Figure 1), which represented 1.66% of the general population representative sample. The median duration of follow-up was 6.2 years (IQR: 3.1–10.6). Follow-up ended due to death for 3,005 (21.8%) patients (mean age at death of 77.8 ± 15.9 years) and 1,660 (12%) patients were lost to follow-up, but the remaining 9,134 (66.2%) subjects were followed until the end of the study period. The study population was predominantly female (56.0%), and the mean age was 55.8 ± 19.4 years. 36.2% of patients were covered by the chronic disease status for psychotic disorders (ALD 23) and 13.2% had free-access-to-care status. The most frequent comorbidity was cardiovascular disease (15.7%). More than half of the patients (63.9%) had received APs in the year before inclusion.

### Antipsychotic prescribing practices

The most frequently dispensed PAP at inclusion was oral or long-acting risperidone, which was the PAP of 2,661 (19.3%) patients. Cyamemazine alone comes as a close second: it was the PAP of 2,581 (18.7%) patients. Olanzapine was the PAP of 1,636 (11.9%) patients, tiapride that of 1,213 (8.8%) patients, and oral or long-acting haloperidol that of 1,036 (7.5%) patients (Table 1). Clozapine was the PAP of 179 (1.3%) patients. In total, after excluding cyamemazine and levomepromazine prescriptions (since their main indications do not concern an antipsychotic effect), the PAP of 4,928 (45.3%) patients was of first generation (FGAP), whereas the one of the 5,942 (54.7%) other patients was of second generation (SGAP). The SGAP/FGAP ratio was thus 1.20, all years combined. Long-acting forms of haloperidol, risperidone, paliperidone, and aripiprazole were the PAP of 339 (2.5%), 308 (2.2%), 52 (0.4%), and 48 (0.4%) patients, respectively (Table 1). In total, long-acting forms constituted the PAP of 747 (5.4%) patients within the study population.

**TABLE 1:** Distribution and use of primary antipsychotics (*n* = 13,799).

Primary AP	*n* (%)
***1st generation***	
Tiapride	1,213(8.8%)
Haloperidol	1,036(7.5%)
Sulpiride	829(6.0%)
Amisulpride	649(4.7%)
Loxapine	362(2.6%)
Pipamperone	170(1.2%)
Zuclopenthixol	156(1.1%)
Chlorpromazine	111(0.8%)
Periciazine	103(0.7%)
Fluphenazine	88(0.6%)
Flupentixol	56(0.4%)
Pipotiazine	54(0.4%)
Pimozide	51(0.4%)
Carpipramine	41(0.3%)
Penfluridol	6(<0.1%)
Perphenazine	3(<0.1%)
Chlorproethazine	0(0.0%)
Droperidol	0(0.0%)
Thioridazine	0(0.0%)
Trifluoperazine	0(0.0%)
***2nd generation***	
Risperidone	2,661(19.3%)
Olanzapine	1,636(11.9%)
Aripiprazole	896(6.5%)
Quetiapine	518(3.8%)
Clozapine	179(1.3%)
Paliperidone (long-acting form)	52(0.4%)
***Other***	
Cyamemazine	2,581(18.7%)
Levomepromazine	348(2.5%)
**Specialty of prescribers**	
General practitioner	6,196(44.9%)
Hospital practitioner (all specialties included)	4,701(34.1%)
Psychiatrist	2,536(18.4%)
Neurologist	110(0.8%)
Neuropsychiatrist	84(0.6%)
Child psychiatrist	3(<0.1%)
**Number of antipsychotics as co-treatment**	
0 molecule	9,073(65.8%)
1 molecule	2,691(19.5%)
2 molecules	1,067(7.7%)
3 molecules	477(3.5%)
4 molecules	233(1.7%)
5 molecules	116(0.8%)
6 molecules and more	142(1.0%)

Almost half (44.9%) of the prescriptions were written by general practitioners, while 34.1% were written by hospital practitioners, and 18.4% by private-practice psychiatrists (Table 1). A majority (65.8%) of patients did not receive any co-dispensing of other antipsychotics, while 19.5% of them were co-treated with one other antipsychotic drug; the remaining 14.7% of individuals were co-treated with at least two additional antipsychotics (Table 1). Table 2 describes the use of the five most frequent PAPs at inclusion. A substantial heterogeneity was found regarding age at inclusion, ranging from 44.2 ± 15.0 years for aripiprazole; 61.5 ± 19.3 years for haloperidol; to 68.6 ± 18.8 years for tiapride. The distribution of doses of all PAPs is displayed in Table 3.

**TABLE 2:** Description of use of the 5 most frequent primary APs (plus cyamemazine).

	Risperidone (*n* = 2,661)		Cyamemazine (*n* = 2,581)		Olanzapine (*n* = 1,636)		Tiapride (*n* = 1,213)		Haloperidol (*n* = 1,036)		Aripiprazole (*n* = 896)	
	Mean (SD)	Median (Q1–Q3)	Mean (SD)	Median (Q1–Q3)	Mean (SD)	Median (Q1–Q3)	Mean (SD)	Median (Q1–Q3)	Mean (SD)	Median (Q1–Q3)	Mean (SD)	Median (Q1–Q3)
Age at inclusion	61.1 (22.2)	62.0 (42−82.0)	52.5 (16.3)	51.0 (41.0−62.0)	49.0 (16.9)	47.0 (36.0−59.0)	68.6 (18.8)	74.0 (54.0−84.0)	61.5 (19.3)	60.0 (46.0−79.0)	44.2 (15.0)	43.0 (33.0−53.0)
Mean time between 2 dispensings, over the first 12 months of follow-up (in days)	36.1 (12.6)	32.6 (28.0−40.4)	34.1 (11.8)	31.5 (27.5−38.3)	30.8 (9.6)	28.6 (26.2−32.9)	34.0 (10.9)	31.5 (28.0−37.3)	35.0 (12.0)	31.6 (28.3−38.1)	31.8 (9.5)	29.8 (26.9−34.5)
Number of dispensed units, over the first 12 months of follow-up	11.7 (7.4)	10.0 (7.0 −14.0)	14.8 (12.0)	12.0 (8.0−18.0)	13.4 (7.7)	13.0 (8.0−16.0)	20.2 (15.1)	16.0 (9.0−27.0)	19.1 (20.2)	13.0 (8.0−23.0)	11.0 (5.6)	11.0 (7.0−13.0)
Time on treatment (in months)	42.5 (41.2)	27.6 (11.5−60.8)	48.8 (44.9)	31.5 (11.0−78.2)	51.8 (49.0)	30.3 (10.7−86.3)	30.7 (33.2)	18.5 (7.1−42.6)	61.1 (52.3)	40.8 (13.8−115.4)	41.3 (41.4)	25.0 (8.3−61.8)

**TABLE 3:** Estimates of the daily dose dispensed (DDD) for the primary antipsychotic (in mg per day).

Primary antipsychotic	N	Mean DDD (SD)	Median DDD	Min-Max DDD
Risperidone	2,661	2.82 (2.25)	2.16	0.18−34.29
Cyamemazine	2,581	56.32 (63.54)	38.46	6.74−1,666.67
Olanzapine	1,636	9.68 (5.84)	8.21	0.89−52.5
Tiapride	1,213	170.76 (122.04)	143.75	19.11−2,500
Haloperidol	1,036	4.35 (4.78)	2.35	0.19−38.62
Aripiprazole	896	10.38 (7.25)	8.7	0.85−105
Sulpiride	829	101.35 (82.05)	86.44	12.3−1,033.86
Amisulpride	649	294.07 (285.96)	175.9	38.96−1,794.39
Quetiapine	518	246.13 (209.85)	168.22	18.52−1,028.57
Loxapine	362	70.93 (82.11)	40.54	6.82−40.54
Levomepromazine	348	61.51 (64.12)	37.64	6.7−37.64
Clozapine	179	183.44 (206.08)	60.96	5.37−809.64
Pipamperone	170	64.06 (38.66)	53.74	11.32−252.63
Zuclopenthixol	156	25.6 (29.29)	15.38	4.48−207.3
Chlorpromazine	111	85.55 (84.13)	62.89	7.81−754.89
Periciazine	103	33.26 (24.86)	29.78	2.06−151.05
Fluphenazine	88	20.65 (55.36)	4.44	1−345.05
Flupentixol	56	10.5 (17.58)	4.1	1−103.23
Pipotiazine	54	7.53 (12.02)	3.81	0.93−66.67
Paliperidone	52	3.11 (1.35)	2.84	0.62−7.14
Pimozide	51	3.21 (2.77)	2.2	0.3−13.04
Carpipramine	41	105.39 (57.85)	86.33	34.78−300
Penfluridol	6	5.94 (2.24)	5.29	2.94−8.89
Perphenazine	3	5.19 (1.91)	5	3.38−7.18

Figures were calculated from the dispensings performed between the inclusion date + 1 month and the date of the last dispensing (over a 12-month period).

### Adherence to antipsychotics

As previously mentioned, %DwT was calculated for the first year of treatment, that is, only for PAPs whose dispensing exceeded 12 months. The PAPs with the lowest mean%DwT, i.e., the drugs to which patients were the most adherent, were paliperidone (9.0%), clozapine (10.6%), and pimozide (10.7%). Conversely, the PAPs with the highest mean%DwT, i.e., those to which patients were the least adherent, were chlorpromazine (26.4%), risperidone (22.7%), amisulpride (21.1%), and cyamemazine (21.0%) (Table 4).

**TABLE 4:** Proportion of days without treatment (%DwT) of each primary antipsychotic over the 12 months after inclusion, among patients with at least 12 months of dispensing (*n* = 9,997).

Primary antipsychotic	N (%)	Mean%DwT (StD)	Median%DwT (Q1–Q3)
Risperidone	1,970(19.7%)	22.7 (21.1)	17.8(2.7−38.3)
Cyamemazine	1,897(19.0%)	21.0 (21.4)	14.6(1.6−34.3)
Olanzapine	1,190(11.9%)	13.0 (19.1)	3.0(0.0−17.8)
Tiapride	806(8.1%)	18.1 (19.5)	9.9(1.4−28.8)
Haloperidol	769(7.7%)	20.2 (19.8)	15.5(2.7−32.1)
Aripiprazole	608(6.1%)	16.5 (20.2)	7.7(0.0−26.0)
Sulpiride	538(5.4%)	18.4 (22.1)	8.5(1.1−30.4)
Amisulpride	453(4.5%)	21.1 (19.9)	15.3(3.3−34.5)
Quetiapine	356(3.6%)	13.4 (19.5)	4.2(0.0−17.8)
Loxapine	273(2.7%)	17.1 (19.8)	9.3(0.8−27.7)
Levomepromazine	250(2.5%)	16.9 (20.6)	6.9(0.6−28.5)
Clozapine	161(1.6%)	10.6 (15.0)	3.3(0.0−17.5)
Pipamperone	137(1.4%)	11.5 (14.8)	3.8(0.0−17.8)
Zuclopenthixol	135(1.4%)	17.5 (17.5)	10.7(2.2−28.8)
Chlorpromazine	87(0.9%)	26.4 (20.5)	20.3(9.6−39.4)
Periciazine	84(0.8%)	17.9 (19.8)	8.7(2.5−32.9)
Fluphenazine	82(0.8%)	15.8 (19.7)	7.3(0.0−26.3)
Flupentixol	52(0.5%)	13.2 (18.4)	3.0(0.0−19.5)
Pipotiazine	51(0.5%)	11.2 (16.1)	3.8(0.0−15.6)
Paliperidone	38(0.4%)	9.0 (13.5)	2.6(0.0−**12**.6)
Pimozide	37(0.4%)	10.7 (16.1)	3.3(0.6−11.8)
Carpipramine	22(0.2%)	20.2 (20.1)	15.1(4.7−26.0)
Penfluridol	1(0.0%)	14.2(−)	14.2(14.2−14.2)

## Discussion

To our knowledge, the APPREAL study is the first to report on the detailed use of chronic antipsychotic treatments at the national level over 10 years, that is, for each drug: the overall rate of prescription, dispensed doses, specialty of the prescribers, estimated level of adherence, as well as the age and main diagnostic codes of the patients. We made two important methodological choices: (1) selecting only patients with at least 3 months of continuous antipsychotic dispensing, effectively excluding punctual prescriptions for emergency or transitory symptoms, and (2) considering cyamemazine and levomepromazine as separate drugs, as their indications essentially consist in treating anxiety and aggressiveness, which are not in line with those of other antipsychotics in France.

Regarding the rates of antipsychotics prescribed, only Kovess-Masfety et al. (9) provided similar national estimates, but only for the year 2015. Moreover, the authors did not define the inclusion criteria as we did, that is, as 3 months of continuous dispensing. Despite this, the authors found results very close to ours, with cyamemazine and risperidone being the two main prescribed drugs. A substantial difference was that olanzapine constituted 5.0% of the antipsychotics prescribed in their study, vs. 11.9% in ours. As we did not explore the longitudinal trajectories of prescribing practices between 2007 and 2017, we hypothesize that the main explanation of this gap is that olanzapine was largely prescribed between 2007 and 2012 in France and much less after that, since it was found to induce significantly more cardiovascular adverse events than other SGAPs (16), thus leading to completely reappraise the risk-benefit ratio of this molecule. All drugs combined, we found a higher SGAP/FGAP ratio (1.20) than Kovess-Masfety and colleagues (1.02), but, as previously explained, we decided to exclude cyamemazine and levomepromazine from the FGAP list, which has certainly led to a higher but probably more exact ratio of SGAP utilization. When compared with international data, France shows some substantial differences in the prescribing practices, in particular regarding quetiapine, which is usually the most prescribed antipsychotic drug worldwide (5), while it constitutes only 3.8% of PAPs in France. This is certainly due to a late marketing of this drug in France, which was approved for reimbursement only in 2011 (17). Overall, the mean age of patients was relatively advanced (55.8 ± 19.4 years). ripiprazole was prescribed in much younger patients than other antipsychotics, including risperidone or tiapride, suggesting sensible differences in the indication and/or in the specialty of the prescribing physicians. Comparisons with other countries reveal wide international disparities in the practices of antipsychotic prescribing. For example, in Australia, a recent study found that 75% of all antipsychotics prescribed were SGAPs and 13% were long-acting drugs (18).

The estimated daily dose dispensed (DDD) and level of adherence were two important and original data in our study. Regarding daily doses, we found relatively low mean DDDs, although these figures should be interpreted in the light of the incomplete level of adherence to drugs, suggesting that the prescribed doses were actually noticeably higher than the doses calculated here, which were only the dispensed doses. Regarding adherence, we found that paliperidone, clozapine and quetiapine were the antipsychotics associated with the highest levels of adherence, whereas risperidone and amisulpride were among those associated with the lowest levels, while being the most prescribed. Cyamemazine was also associated with a low adherence, but this might be due to the conditions of use of this drug, which can be more easily used as an as-needed treatment, since it targets anxiety or irritability. Regarding other oral antipsychotics, our results are in line with other studies, which found that clozapine was the antipsychotic drug with the highest level of adherence (19), and that olanzapine, quetiapine, and aripiprazole were associated with low rates of treatment discontinuation, when compared to other antipsychotics (20). However, our study is the first to confirm this in real-life French data. Furthermore, our study provides important findings regarding the global use of long-acting forms of antipsychotics, which is quite low (5.4%), especially since at least one third of all prescriptions in our study were associated with psychotic disorders, and long-acting forms should theoretically be systematically proposed to all patients with psychotic disorders (21). Overall, our results are in line with previous studies showing that clozapine and long-acting forms are the types of antipsychotics associated with the highest rates of adherence (22).

Another interesting and original finding of our study is the rate of multiple antipsychotic prescriptions. More than one third of French patients received at least once more than one antipsychotic drug, while almost 15% of them simultaneously received at least three concurrent antipsychotics or more. International guidelines on diseases such as schizophrenia or dementia all recommend to only exceptionally combine antipsychotic treatments (23, 24). For example, in schizophrenia, antipsychotic polypharmacy should be considered only in resistant forms and after ineffective clozapine treatment (25). However, such combinations seem to be relatively frequent in real life, as the rate of association was found to reach 25% in the exhaustive Swedish population (26), between 20 and 30% in the Italian population (27), and 13% in the U.S. in 2003 (28). Polypharmacy of antipsychotics was even found to reach 43% among inpatients (29). French figures are particularly high and suggest the need for further health policy initiatives. Regarding the specialty of prescribers, we showed that general practitioners were by far the most frequent prescribers of antipsychotics (44.9%), in particular when compared with office-based psychiatrists (18.4%); prescriptions from neurologists (0.6%) were marginal. However, these figures should be interpreted with great caution because hospital physicians represent 34.1% of antipsychotic prescribers, but it is not possible to identify their exact specialty in the EGB.

### Strengths and limitations

Results are based on high-quality claims data, which include comprehensive information on treatments and on the use of reimbursed healthcare resources in a representative sample of the French population. Additionally, as the French health insurance system is accessible to all, the data covers all types of populations, regardless of their age, social condition, or economic resources. Medico-administrative databases are increasingly used as indicators of adherence and persistence because they have the advantage of being able to study large populations over specific periods of time, unaffected by recall bias. Such analyses can also be carried out repeatedly and over extended periods of time, as a monitoring tool.

However, diagnoses were only available in case of ALD status or hospital admission. In addition, the distribution of over-the-counter drugs, the prescribed daily doses, the duration of prescriptions, and unfilled prescriptions are not available. Another limitation is that the EGB does not contain data on drugs dispensed during hospital stays (except for very costly medications). The EGB only includes hospitalizations in establishments that carry out medical, surgical, obstetrical, dental, and ambulatory activities, as well as cancerology. It therefore does not include psychiatric activities, follow-up and rehabilitation care, or home hospitalization. This means we were unable to identify patients who received a dispensing of APs in a psychiatric hospital (during an outpatient consultation or hospitalization) or in a nursing home (with an internal pharmacy). We were also unable to identify diagnoses coded in psychiatric hospitals. Finally, compliance was only assessed using the percentage of dispensings and biological measures would have been more reliable, but they are not undertaken in routine clinical practice and are therefore not available within the EGB.

## Conclusion

Overall, antipsychotic prescribing practices were very heterogeneous and displayed both similarities and differences, when compared with other countries. In particular, the use of cyamemazine was widespread in France, even though its indications are generally not for psychotic symptoms. The use of FGAPs remained high, while that of long-acting antipsychotics was low. Polypharmacy was particularly important, suggesting the need to better inform prescribers about the iatrogenic risks resulting from such practices. The levels of patient adherence to the different drugs were relatively in line with similar international investigations, suggesting that clozapine, olanzapine, and quetiapine are the drugs associated with the highest levels of adherence.

## Data availability statement

The datasets presented in this article are not readily available because data belong to the French Insurance Database. Requests to access the datasets should be directed to manon.belhassen@pelyon.

## Ethics statement

The studies involving human participants were reviewed and approved by the Commission Nationale Informatique et Libertés. Written informed consent for participation was not required for this study in accordance with the national legislation and the institutional requirements.

## Author contributions

BR, MBel, EV, and FD designed the study. FD, MBér, FJ, and MBel extracted and analyzed the data. BR, FD, NG, GB, LC, and MBel provided regular comments on the results and suggested further analyses. BR and MBel wrote the first draft of the manuscript. All authors have read and approved the final draft of the manuscript.
